# Reflex Lesions of the Mouth, Associated with Pregnancy

**Published:** 1882-10

**Authors:** Joseph Richardson


### THE DENTAL REGISTER.

VOL. XXXVI.] OCTOBER, 1882. [No. 10.

### COMMUNICATIONS.

### Reflex Lesions of the Mouth, Associated with Pregnancy,

BY JOSEPH RICHARDSON, M.D., D.D.S.

At a late meeting of the Indiana State Dental Association, it was my privilege to read before that body a paper entitled "Correlated Diseases of the Teeth and Ear." (See August number of the DENTAL REGISTER.) The purpose of the paper was to present some ascertained facts in regard to the reflex nervous relationship existing between the teeth and the various structures of the auditory apparatus, and to account for the manner in which not only functional but nutritive or trophic diseases of the latter occurred as a sequence of irritation having its source primarily in the teeth or gums.

Researches directed to the ascertainment of sympathetic relationships of other organs, as the eye, brain, stomach, uterus, etc., would probably reveal them also as so many areas correlated in like manner as the ear to primary disorders of the organs of the mouth.

It is not my purpose, however, to consider farther, in this place, reflex diseases of organs or regions more or less remote from the mouth, but to reverse the order of inquiry, and trace if possible the sympathetic dependency of certain disorders of the teeth upon functional disturbances or structural lesions primarily located in some remote part of the general organism; premising, that a better understanding of the laws of reflex action, a more familiar and exact acquaintance with nerve function, and a clearer insight into the correlated areas existing between widely separated regions, would lead to more rational and trustworthy views of the etiology of many sympathetic disorders of the buccal cavity, the origin and nature of which are, as yet, measurably a sealed book to us.

In the discussion of my subject, I shall, in order to confine myself within reasonable limits, consider only certain abnormal or pathological states of the oral cavity frequently associated with pregnancy.

It might be thought that, as utero-gestation is essentially a physiological act, it would hardly be competent to give rise to such irritation as would establish sympathetic disorders of the teeth, and this would doubtless be true if the uterine functions during this period were always normally performed. But the simple fact that the reproductive act is ordinarily, a *conscious* one, leads obviously to the conclusion of functional derangement, for the senses take no cognizance of the normal processes of organic life, and hence no one, in perfect health, through the medium of common sensation, is supposed to have any mental perception of a brain, stomach, liver, or uterus, or of their functional processes. The very uniformity of the oral affections coincident with pregnancy is, in itself, presumptive evidence of perturbed uterine function more or less severe and persistent.

Before proceeding to point out the nervous relationship between the uterus and the oral cavity, it would seem pertinent to examine briefly the generally accepted theory in explanation of certain abnormal states of the organs of the mouth associated with gestation.

The prevailing view, I believe, is that the tendency to more rapid decay of the teeth characterizing this period is due to the increased consumption of lime salts incident to fœtal growth and development, and that this superadded draft upon the calcific elements of the maternal circulation, by diverting the lime salts ordinarily appropriated by the teeth in the processes of repair, induces a nutritive lesion characterized by deficiency of ossific deposits, and consequent structural impairment—a condition rightly supposed to favor accelerated decay.

This theory would seem to be predicated on the assumption that the bone-producing elements of the ingesta are, in the unimpregnated state, largely if not wholly appropriated by the tissues into which they enter as a component—that there is not ordinarily, any adequate excess to meet the exigency under consideration, and that when such exigency occurs, it becomes, as between the necessities of mother and child, a simple question of *divide.*

Now, aside from comparatively infrequent cases of tissue starvation consequent on pronounced deficiency of the bone-producing elements in the food, it would seem reasonable to suppose that there is, ordinarily a prevailing excess of calcific matter in the maternal circulation adequate for all the contingent needs of both mother and offspring. If this were not so—if, in the unimpregnated state, there were but a simple sufficiency without excess, the fœtus would suffer in common with the mother, and we should expect, whenever the demand for increased supply occurred, to find, not only structural impairment of the mother's teeth during the gestative period, but, as the result of imperfect ossific deposits during the period of fœtal evolution, osteological malformations a common characteristic and a common inheritance of the human race—conditions, the existence of which has no warrant in facts or observation.

If this theory of diversion, or robbing Peter to pay Paul, is, as I believe it to be, neither rational, nor in accordance with any just interpretation of the laws of nutrition, neither is it acceptable in explanation of other phenomena associated with utero-gestation. It does not account for the distressing and oftentimes violent pains associated with teeth but slightly decayed, and often with those of unbroken structure, nor does it in any manner explain the presence of the various neuralgic affections of neighboring parts coincident with impregnation.

We must look farther, then, for some more rational solution of the problem of causation as it relates to the sympathetic oral phenomena associated with the reproductive processes.

A more plausible and defensible theory, I believe, would assign to these phenomena the character of reflex lesions, functional or structural. To justify this conclusion, I shall first endeavor to ascertain if the organs of the mouth constitute an area correlated to the uterus through the medium of the sympathetic system of nerves, and then consider in what manner the vaso-motor fibres of that system operate as factors in the production of the oral phenomena which I have characterised as reflex lesions.

It is important to remember in this connection the essential fact now broadly recognized by modern physiologists, namely, that the vaso-motor fibres of the great sympathetic system of nerves are largely distributed to the blood vessels, and that their peculiar and distinctive office is to control and regulate the caliber of the vessels to which they are distributed, and consequently the blood-supply to any given part.

Bearing this central fact in mind, the next inquiry is, do the teeth constitute an area correlated to the uterus through the medium of the sympathetic system? The relationship is, I think, sufficiently obvious, and may be traced as follows: The blood-supply to the teeth is derived from the external carotid through its branches, the inferior dental, and alveolar branch of the internal maxillary arteries. The vaso-motor nerves controlling the caliber or tonicity of these vessels are derived immediately from the external carotid plexus of the sympathetic—a plexus derived from the superior cervical ganglion of the spinal sympathetic.

The uterus and its appendages and vaginal walls are supplied with nerves from the pelvic plexus of the sympathetic, bringing these several parts into immediate relation with the spinal sympathetic system.

Thus it will be seen that any tissue impression having its source in the uterus or associated structures, would be transmitted by way of the afferent vaso-motor fibres to the vaso-motor centre in the medulla, and returning by the efferent fibres in reflex relationship with the former, would reach the carotid plexus through the superior cervical ganglion. As the branches of the external carotid artery, to which this plexus is distributed, supply the teeth with blood vessels, it is not difficult to see how the teeth of the superior and inferior maxilla become an area correlated to the uterus through the medium of the carotid plexus of the sympathetic system.

The correctness of this view of the relationship of the regions under consideration being granted, it only remains to ascertain how, through the action of the vaso-motor nerves, nutritive lesions or reflex functional disturbances in connection with the teeth may occur as a sequence of irritation having its origin in the uterus, its appendages, or vaginal walls.

Dr. Charles H. Burnett, commenting on reflex diseases of the ear, says: "It may be stated in a general way that *the effect of any irritation in a vaso-motor nerve-tract may be to excite vessel-dilatation through diminished inhibitory nerve-power, in a correlated area*," while Dr. Woakes, of London, affirms the fact of dilitation of the vessels as a constant condition following reflex irritations.

Accepting this statement as an ascertained physiological fact, we should have, as the result of any irritation having its source in the gravid uterus or its associated parts, impaired tonicity of the pulp-vessels, manifesting itself in dilatation and consequent congestion or hyperæmia. If the disturbance in the pelvic region were such only as to produce but slight irritation, the reflex lesion of the pulp would manifest itself as a so-called sympathetic neuralgia consequent on pressure of nerve fibres by the engorged and distended blood-vessels. Sympathetic tooth-ache manifesting itself in teeth of sound as well as unsound structure, is very commonly associated with pregnancy, and cannot be rationally accounted for, I apprehend, except in the manner here indicated. Ordinarily, the exciting cause would be insufficient to induce more than functional disturbance in the pulp, but it is easy to conceive how such reflex irritations of sufficient intensity and persistence might provoke blood-stasis, inflammation, and disorganization or death of the pulp.

Let us next apply the conclusions of this paper in explanation of the phenomena of softening and more rapid decay of the teeth during the period of gestation. The lesion is, I think, clearly a reflex one. Anything like prolonged hyperæmia of the vessels of the pulp, must, by altering or diminishing the blood-supply, seriously interfere with the nutritive processes concerned in the reparation of wasting tissues. The consequence of such interference with the normal functions of the pulp, in so far as its vessels are concerned in the nutrition of the tooth, would be in the direction of degeneracy or retrograde metamorphosis of tooth structure. This would imply, we may suppose, defective assimilation of ossific pabulum for purposes of repair, and certainly diminished vital resistance to the action of agents operative in producing solution of the lime salts. We should have, consequently, from the operation of these causes, a tendency to general softening, with hyper-sensitiveness, and a greatly increased rapidity of decay. These are frequent coincident complications of pregnancy, are always more or less distressing, and often involve the loss of valuable organs. The subject is, therefore, one of great interest to us as oral specialists.

Whether the views here advanced as to the cause and nature of these lesions are correct or not, they are submitted to the candid and impartial criticism of the profession.

